# Maternal gene expression in Atlantic halibut (*Hippoglossus hippoglossus *L.) and its relation to egg quality

**DOI:** 10.1186/1756-0500-3-138

**Published:** 2010-05-24

**Authors:** Maren Mommens, Jorge MO Fernandes, Teshome T Bizuayehu, Sylvie L Bolla, Ian A Johnston, Igor Babiak

**Affiliations:** 1 Faculty of Biosciences and Aquaculture, Bodø University College, N-8049 Bodø, Norway; 2 Scottish Oceans Institute, School of Biology, University of St. Andrews, East Sands, St. Andrews, Fife, KY16 8LB, UK

## Abstract

**Background:**

The commercial production of Atlantic halibut (*Hippoglossus hippoglossus *L.) suffers from a major bottleneck due to the low success of producing juveniles for on-growing. Atlantic halibut females are routinely hand-stripped and incorrect timing of stripping can result in low quality eggs due to post-ovulatory aging. Post-ovulatory aging leads to compositional changes in eggs that include maternally provided proteins and RNAs. There have been few studies of the maternally provided mRNA transcripts that control early development in commercially important fish species. The present study aimed to study maternal gene expression in Atlantic halibut and its relation to egg quality parameters including blastomere symmetry and hatching success.

**Results:**

A maternal EST library containing 2341 sequences was constructed by suppressive subtractive hybridisation. Thirty genes were selected for expression studies; 23 novel genes and 7 genes with documented roles in early development. The expressions of twenty-one selected genes were measured by qPCR from fertilization to the 10-somite stage. Three genes were identified as strictly maternal genes that were expressed until the start of gastrulation; *askopos *(*kop*), *si:dkey-30j22.9 *(Tudor family member), and *Tudor 5 protein *(*Tdrd5*). The expressions of 18 genes at the 8-cell stage were correlated with egg quality parameters. The majority of genes showed either no or very minor correlations with egg quality parameter. However, two genes correlated positively with hatching success (*r*> 0.50, HHC00353: *r *= 0.58, *p *< 0.01; HHC01517: *r *= 0.56, *p *< 0.01) and one gene (HHC00255) was negatively correlated with the percentage of normal blastomeres (*r *= -0.62, *p *< 0.05).

**Conclusions:**

During this study we have related maternal levels of gene expression to hatching success in fish. Poor hatching success was not correlated with a general decrease in transcript abundance but with low transcript levels of some specific genes. Thus, the molecular mechanisms leading to low Atlantic halibut egg quality cannot be entirely explained by post-ovulatory aging.

## Background

Atlantic halibut (*Hippoglossus hippoglossus *L.) is considered a valuable species for cold water marine fish farming, but current production suffers from a bottleneck during fry production for on-growing. Variable egg quality is a common problem in commercial fish farming [1,2]. Good quality eggs have been defined as eggs exhibiting high survival rates at fertilization, larval hatching and larval first feeding [3]. Poor egg quality leads to major problems during the early production stage, including high embryonic and larval mortality and body malformations [1,4]. Domesticated Atlantic halibut can release eggs naturally, but they are usually not fertilized [5]. Atlantic halibut females, used as commercial broodstock, are typically between 30-100 kg in size. For seed production, males and females are routinely hand-stripped for gametes and eggs fertilized in the hatchery. Atlantic halibut is a batch spawner and can release up to 10 batches off eggs at intervals of 2 - 3 days. It is necessary to carefully monitor individual females since egg quality is only optimal for some hours after ovulation [6]. Incorrect timing of stripping can result in low egg quality due to post-ovulatory aging. Post-ovulatory aging leads to a series of morphological and compositional changes in eggs including maternal mRNA concentrations [7].

Egg quality can be affected by several biotic and abiotic factors, such as female size, genetic influences and broodstock nutrition and management [2]. How early gene regulation influences egg quality is little studied in fish and especially farmed fish [8]. High embryonic mortality rates are often observed within several days after fertilization in Atlantic halibut [9]. During this period, embryonic development is initially regulated by maternally provided mRNAs, followed by a transition to the zygotic genome, the so called maternal zygotic transition (MZT). Even though maternal mRNAs get degraded during MZT, they play an important role in initiating processes crucial to patterning the developing fish embryo such as axis formation, specification of somatic tissue lineages and germline [10,11]. The MZT is a progressive process and some maternal-zygotic genes are expressed both before and after the MZT [12]. Maternal-zygotic and finally zygotic gene expression regulates the later parts of axis formation and organogenesis during fish embryonic development [13].

Genetic studies of early stages of Atlantic halibut have concentrated so far on embryonic somite formation or larval stages [14,15]. With the exception of a 2-cell cDNA library [16], previous libraries were constructed from post-embryonic stages.

In the present study, we used suppression subtractive hybridisation to create an expressed sequence tag (EST) library between fertilized eggs and 10-somite stage embryos to find maternal genes in Atlantic halibut. Totally, 30 genes were selected for expression studies; 23 novel genes and 7 genes with documented roles in early development (Additional file 1). From these, 21 genes were screened for their relative expression from fertilization to the 10-somite stage to verify their expression. To study how maternal transcripts influence egg quality, expression levels were correlated to fertilization and hatching percentages in 29 different egg batches. In 13 egg batches, expression of the candidate quality markers was also correlated with the percentage of eggs with normal blastomere symmetry during early cleavages.

## Methods

### Sampling and husbandry

Egg samples were collected from Atlantic halibut (*Hippoglossus hippoglossus *L.) females at two different locations in Norway. Four batches were sampled from a commercial farm (Risørfisk AS, Risør, Norway) in 2007 and 25 batches at Bodø University College, Bodø, Norway in 2006, 2008 and 2009 (Additional file 2). All eggs were fertilized *in vitro *with pooled sperm from two random males. The females at Risørfisk AS consisted of fish between 30-40 kg, fed EWOS Premix (EWOS, Bergen, Norway) and kept under natural photoperiod conditions. At Risørfisk AS, eggs were incubated in large scale in 280 liter incubators at salinity between 33-35 ‰ and temperature between 6.2 -6.4°C. Egg samples were taken at the following stages: fertilized eggs (FE); 8-cell stage (8CS), 8 hours past fertilization (hpf), 16 cell stage (16CS), 12°hpf; blastula (BL), 45 hpf; germ ring (GR), 82 hpf; 25% epiboly (25EP) 96 hpf; 50% epiboly (50EP), 117 hpf; and 10 somite stage (10SS), 142 hpf. Samples were wrapped in tinfoil and snap-frozen in liquid nitrogen. Embryos were incubated in triplicates (approximately 100 eggs per replicate) in Petri-dishes at temperature between 5.0 -5.4°C overnight to estimate relative fertilization (%) at the 8-cell stage. Relative hatching (%) was estimated by daily volumetric measurements of dead eggs from incubators from fertilization until hatching. At Bodø University College, in 2006, some females were kept under natural photoperiod conditions and fed herring for human consumption (winter herring) stuffed with Fish Breed-M (INVE Aquaculture NV, Dendermonde, Belgium) (1:1). Other females, kept under photoperiod advanced of approximately 1 month, were fed Fish Breed-M. Eight batches were incubated in large scale incubators, and their performance was estimated under similar conditions as at Risørfisk AS. In 2008 and 2009, all females were kept under natural photoperiod and fed Fish Breed-M. Embryos were sampled at the 8-cell stage and snap-frozen as described above. Seventeen batches were incubated in small scale Petri dishes, as described above, at 5.5 ± 0.5°C in 33 ‰ filtered seawater, added Penicillin-Streptomycin-Neomycin solution (100 Units Penicillin, 0.25 mg Streptomycin and 0.5 mg Neomycin per ml, Sigma, St. Louise, Mo, USA). Relative fertilization was estimated at 8-cell stage. The water in the Petri dishes was changed after GR stage. To estimate relative hatching, dead eggs were counted and removed every second day until hatching. For 13 batches sampled at the Bodø University College, blastomere symmetry was estimated at the 8-cell stage on 30 eggs. Regular blastomere cleavage and abnormalities in blastomeres were estimated according to Shields et al [17].

### RNA extraction and cDNA synthesis

Total RNA for all samples were extracted according to the Tri reagent method (Sigma, St-Louise, MO USA) using QIAazol (Qiagen, Nydalen, Sweden). Total RNA was treated with the gDNA wipeout buffer supplied with the QuantiTect reverse transcription kit (Qiagen) to remove traces of genomic DNA contamination. RNA concentration was quantified using the Nanodrop spectrophotometer (Nanodrop Technologies/Saven Werner, Kristiansand, Norway).

Blunt-ended cDNA fragments for the substraction were produced with a SMART PCR cDNA synthesis kit (Clontech, Saint-Germain-en-Laye, France) and digestion with *Rsa *I. The digested cDNA was then purified with a QIAquick PCR Purification kit (Quiagen) and quantified using Nanodrop.

### Suppressive subtractive hybridization

A forward subtractive library between fertilized eggs (maternal) and 10 somite stage (zygotic) embryos was created by suppressive subtractive hybridization (SSH) using the PCR-select cDNA subtraction kit (Clontech) [18]. Blunt-ended cDNA fragments from fertilized eggs (FE) were used as a tester while the fragments from the 10 somite stage (10SS) were used as a driver. The ligation step was optimized for the halibut samples using an Atlantic halibut specific primer for *β2-tubulin *(Fwd: TACAATGAGGCTTCAGGTGG, Rev: TCCCTCTGTGTAGTGACCCTTG) using an annealing temperature of 65°C and amplifying a product size of 134 base pairs. The subtracted PCR product for the fertilized eggs was cloned with the TOPO TA Cloning Kit (Invitrogen, Paisley, UK) and random clones were picked for sequencing. Insert checks were carried out by PCR with 1 μl of colony template mixed with 20 μl of reaction mix (dNTPs, 2 mM), PCR buffer (10 x), T3 primer short (10 μM) (5' ATTAACCCTCACTAAAG 3'), T7 primer short (10 μM) (5' AATACGACTCACTATAG 3'), Taq DNA Polymerase (GE Healthcare, Nydalen, Norway) and MilliQ water. The PCR involved an initial denaturation step at 96°C for 2.5 min followed by 36 amplification cycles: 96°C for 20 sec., 48°C for 30 sec, and 72°C for 1 min with a final extension at 72°C for 5 min. 5' end sequencing PCR sequencing reactions with T3 primer (5' AATTAACCCTCACTAAAGGG 3') were performed using the ABI prism Big Dye Terminator Sequencing Kit (PE Applied Biosystems, USA) BetterBase (Web Scientific, Crewe, UK). The sequencing reaction comprised an initial denaturation at 96°C for 1 min and 25 cycles at 96°C for 10 sec and 60°C for 3 min and DNA was send for sequencing at the Oxford University sequencing facility with an ABI 3700 capillary sequencer (PE Applied Biosystems, USA).

### Sequence processing and bioinformatics analysis

The raw sequence trace data were processed by the EST analysis pipeline developed by the Natural Environment Research Council-Environmental Genomics Thematic Programme Data Centre (NERC-EGTDC; University of Edinburgh, UK). The electrophoregrams were first analyzed by trace2dbEST (accessible through http://www.nematodes.org/bioinformatics/trace2dbEST/) which processes raw sequencing chromatograph trace files from EST projects into quality-checked sequences. High quality sequences required > 150 high quality bases, based on signal strength, peak shape and peak local environment [19]. Sequences were submitted to dbEST http://www.ncbi.nlm.nih.gov/projects/dbEST/ jointly with their BLAST-based preliminary annotation. PartiGene [20] was then used to cluster the sequences and contig assembly. The non-redundant clusters were submitted to BLASTX similarity searches against the non-redundant (nr) protein database at the National Center for Biotechnology Information (NCBI, http://blast.ncbi.nlm.nih.gov/Blast.cgi). The EST annotation tool Blast2GO [21] was employed for gene ontology [22], enzyme code annotation and pathway mapping with the Kyoto Encyclopedia of Genes and Genomes (KEGG; [23]).

### Relative gene expression by quantitative-real time PCR (qPCR)

Twenty-one genes from the maternal cDNA library were chosen for screening during embryonic development (Additional file 1). Nine genes with three different patterns of relative gene expression, maternal, maternal-zygotic and constant, were further selected to test if they were related to egg quality. Their gene expression was estimated in twenty-nine Atlantic halibut batches at the 8-cell stage. In addition, 9 separate random genes from the maternal cDNA library were chosen for the same analysis. Whenever possible, primers were designed across the most conserved splice junctions. All gene specific primers crossed at least one intron/exon border containing both donor and acceptor sites, in order to avoid amplification of any contaminating genomic DNA. Primer pairs for qPCR amplification were designed manually and screened for hairpins, homo- and cross-dimers using *Netprimer *http://www.premierbiosoft.com/netprimer/ (Additional file 3). To confirm that the right product was amplified, a qPCR was performed on pooled cDNA for each primer pair. The different products were sequenced directly for additional verification. Each sample was checked for genomic DNA contamination by running a qPCR with RNA treated with gDNA whipeout buffer (Qiagen). Gene amplifications by qPCR were performed with a LightCycler^® ^480 thermocycler (Roche, Basel, Switzerland). Each 10 μl reaction in a 96-well plate comprised 4 μl of 70 x diluted cDNA template, 1 μl of each primer pair at 5 μM and 5 μl of QuantiTect SYBR Green containing ROX as reference dye (Qiagen). After an initial denaturation step of 15 min at 95°C, 45 cycles of amplification were performed according to the following thermal cycles: denaturation for 15 s at 94°C, annealing for 20 s at 60°C and extension for 20 s at 72°C. Fluorescence data were acquired during this last step. A dissociation protocol with a gradient from 65 to 97°C was used to investigate the specificity of the qPCR reaction and the presence of primer dimers. All samples were run in duplicate along with minus reverse transcriptase, no template and a positive plate controls. Five-point standard curves of a 5-fold dilution series (1:2-1:16) from pooled cDNA were used for PCR efficiency calculation. To assess suitable reference genes for the qPCR studies the known reference genes *elongation factor 2 *(*Eef2*), *β2-tubulin *(*Tubb2*) and *β-Actin *(*Actb*) were tested [24] (Additional file 4). In addition, genes HHC01138, HHC1517 and HHC00353 were selected to test their suitability as reference genes based on their stable quantification cycles (Cq). GeNorm [25] was used to assess the most suitable reference genes. Primers for qPCR were designed as described above for the genes of interest (Additional file 4). HHC01517 and HHC00353 were selected as the most suitable reference genes for developmental stages, FE to 10SS (Additional file 5, Figure A and additional file 6); where as *β2-tubulin *(*Tubb2*) and *β-actin *(*Actb*) were selected for the 8-cell stage (Additional file 5, Figure B). According to GeNorm these genes were rather stable and, therefore, expression profiles were normalized assuming a similar quantity of total RNA for all studied stages. Nevertheless, there might be some variation in total RNA levels per embryo during early development.

### Data analysis and Statistics

Class discovery was done with the clustering software available on the Gene Expression Pattern Analysis Suit 4.0 (GEPAS, http://gepas.bioinfo.cipf.es/). Relative gene expression data was log transformed and standardized against fertilized eggs (FE). Clustering was performed according to the single-linkage method and weighted pair group method (WPGMA) using Pearson's correlation coefficient (*r*) as distance measurement.

Statistical analysis was done using SPSS 15.0 (SPSS Inc., Chicago, IL, USA). After a log transformation of the relative expression all variables complied with the assumptions for a one-way ANOVA. When significant differences were identified a supplementary Tukey's *post-hoc *test was performed to investigate differences between developmental stages. Statistical significance was established at *p *< 0.05.

Pearson's correlation coefficient was estimated between gene expression and egg batch performance parameters. Fertilization, hatching and symmetry percentages were *arc sin square roots *transformed before correlation analysis [26]. Statistical significance was established at *p *< 0.05. Differences in gene expression between eggs with low hatching rates (< 40%, *n *= 9) compared to eggs with high hatching rates (> 60%, *n *= 15) were estimated using Welch's t test. Statistical significance was established at *p *< 0.05.

## Results

### Characterization of EST library

An EST library was constructed by suppressive subtractive hybridization, subtracting cDNA from fertilized eggs against the cDNA from 10-somite stage embryos. A total of 4592 clones were randomly picked and sequenced from their 5' end. After screening for vector and *E. coli *sequences, only ESTs longer then 150 base pairs were chosen for further analysis. The analysis resulted in 2341 high quality EST sequences with an average length of 344 base pairs that were submitted to the EST database dbEST (http://www.ncbi.nlm.nih.gov/projects/dbEST/, GeneBank accession numbers FK701051-FK703391) together with their BLAST-based preliminary annotations. Grouping the EST sequences into non-redundant cluster with PartiGene resulted in a total of 1064 putative gene clusters. The overall redundancy for the maternal library was 2.7, with 77% of the putative genes being represented by only one EST. Subjecting non-redundant clusters to BLASTX similarity searches against the non-redundant (nr) protein database at NCBI resulted in significant matches for 26.5% of the clusters (Table 1). In addition, 28% of the cluster had matches against unnamed and hypothetical protein products. The largest gene clusters with significant hits corresponded to structural proteins or metabolic enzymes such as mitochondrial genes encoding cytochrome *b *and cytochrome oxidase subunits and the nuclear genes encoding myosin heavy chain. The remaining 45.5% of the assembled clusters did not have significant matches against the nr protein database.

**Table 1:** Most abundant EST clones in maternal cDNA library from Atlantic halibut (*Hippoglossus hippoglossus *L.).

Gene	Number of sequences	Number of clusters	Transcript abundance (%)
*Unknown genes*			
Genes with no significant hits	1064	395	45.5
Unnamed protein products	508	218	21.7
*Tetraodon nigroviridis*			
Hypothetical proteins *Danio rerio*	134	62	5.7
Hypothetical proteins others (Gallus, Oryzia, Xenopus, Mus, Homo)	14	10	0.6
*Mitochondrial genes*			
Cytochrome b	83	3	3.5
Mitochondrial hypothetical 18 K protein-goldfish	63	63	2.7
Cytochrome c oxidase subunit I	21	11	0.9
Cytochrome c oxidase subunit II	11	5	0.5
Cytochrome oxidase subunit III	8	3	0.3
*Nuclear genes*			
Myosin heavy chain	38	10	1.6
skeletal muscle fast troponin T	19	5	0.8
Creatine kinase	19	7	0.8
Parvalbumin	18	2	0.8
Tropomyosin alpha chain	11	2	0.5
Septin 7	10	1	0.4
Caprin family member 2	9	3	0.4
Odorant receptor	9	2	0.4
Skeletal muscle alpha actin 1	8	4	0.3
RNA binding protein with multiple splicing 2	8	2	0.3
Cyclin A2	8	1	0.3

BLASTX search significance level was set to E value < 0.001.

**Figure 1:**
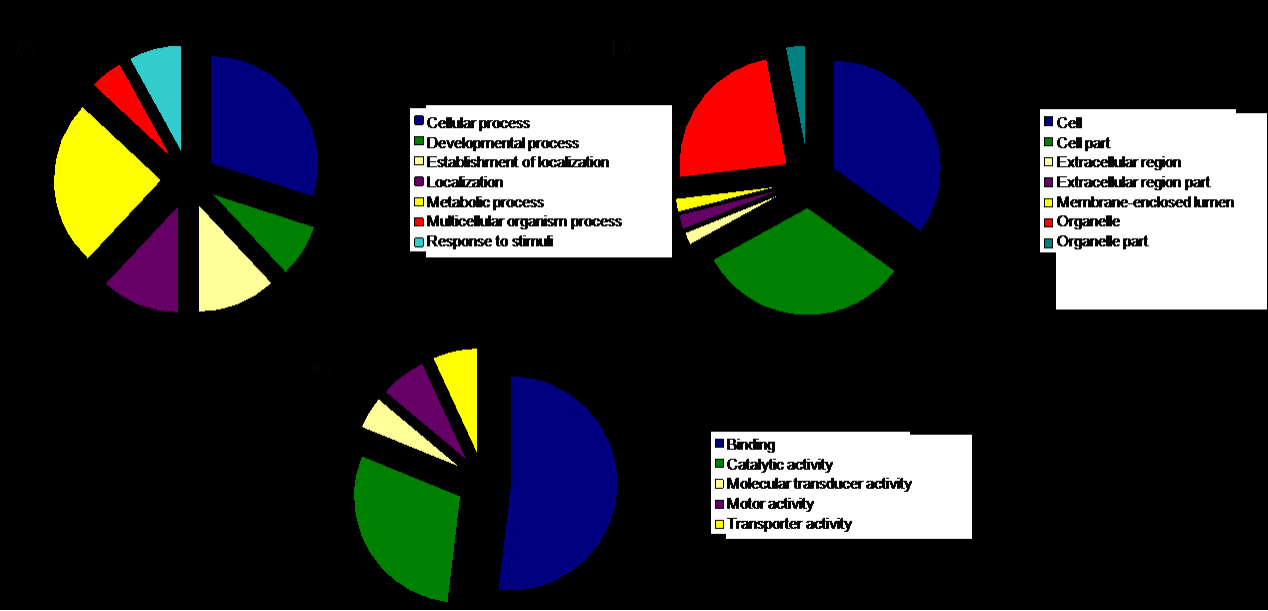
**Gene classification based on Gene Ontology**. A) Biological process. B) Cellular component. C) Molecular function.

Annotation in Blast2GO against the Gene Ontology (GO) database resulted in 699 clusters being annotated with a total of 3956 GO terms at a mean GO level of 5.07. Furthermore, 261 enzyme codes were mapped to 196 sequences. The annotated sequences were grouped into different classes of ontology according to the GO terms, as shown in Figure 1. Most of the genes involved in biological processes were part of metabolic and cellular processes. Half of the annotated genes were classified as genes with the function of binding, followed by the function of catalytic activity. Searching against the Kyoto Encyclopaedia of Genes and Genomes (KEGG) pathways resulted in the annotation of 169 of the clusters representing 83 different pathways http://www.genome.jp/kegg/pathway.html. The thirteen most represented pathways are shown in Table 2.

**Table 2:** The most commonly represented KEGG pathways of maternal sequences from Atlantic halibut (*Hippoglossus hippoglossus L*.).

Pathway	Number of clusters	Transcript abundance (%)
Urea cycle and metabolism of amino groups	8	4.7
Glycan structures	6	3.6
Purine metabolism	6	3.6
Glycolysis/Gluconeogenesis	5	3.0
Drug metabolism-other enzymes	5	3.0
Pyruvate metabolism	5	3.0
Pyrimidine metabolism	4	2.4
Lysine degradation	4	2.4
Tryptophan metabolism	4	2.4
Butanoate metabolism	4	2.4
Pentose phosphate pathway	4	2.4
Carbon fixation	4	2.4
Beta-Alanine metabolism	4	2.4

### Screening of relative gene expression during early embryonic development

**Figure 2:**
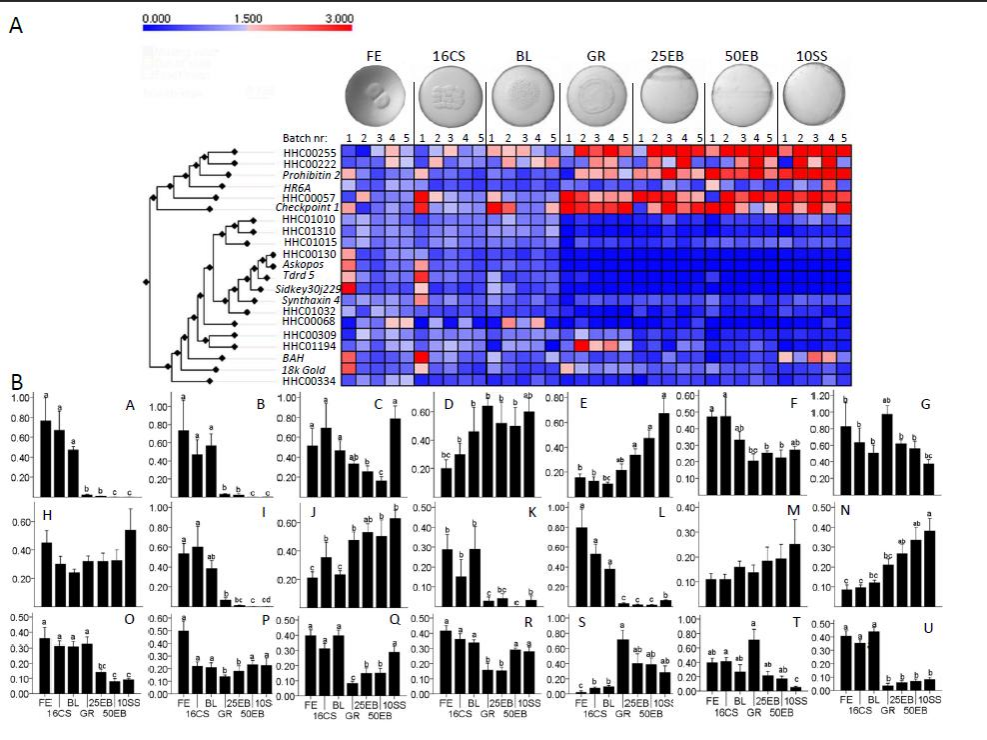
**Gene class discovery**. A: Clustering of genes, according to their relative gene expression during early embryonic development in Atlantic halibut. Genes were clustered using the single-linkage method with Pearson correlation coefficient as distance measurement. The developmental stages were fertilized eggs (FE); 8-cell stage (8CS), 8 hours past fertilization (hpf); 16 cell stage (16CS), 12 hpf; blastula (BL), 45 hpf; germ ring (GR), 82 hpf; 25% epiboly (25EP) 96 hpf; 50% epiboly (50EP), 117 hpf; and 10 somite stage (10SS), 142 hpf (*n *= 5). Data was standardized against the first stage, fertilized eggs (FE). Colour bar indicates relative gene expression in relation to FE. Red colour shows up-regulation and blue colour down-regulation in relation to FE. Reference genes are not included. B: Relative gene expressions of selected genes from fertilization to 10 somite stage. Expression pattern of a. *Askopos*, b. *Si:dkey-30j22.9*, c. *BAH*, d. *Checkpoint 1*, e. *Prohibitin 2*, f. *Synthaxin 4*, g. *18 k hypothetical goldfish protein*, h. *HR6A*, i. *Tudor 5 protein*, j. HHC00057, k. HHC00068, l. HHC00130, m. HHC00222, n. HHC00255, o. HHC00309, p. HHC00334, q. HHC01010, r. HHC01015, s. HHC01032, t. HHC01194, u. HHC01310. Error bars indicate the standard deviation (*n *= 5).

Twenty-one genes were selected from the library for screening of their relative expression during early development, 14 novel genes and 7 genes with documented roles in early development (Additional file 1). Relative gene expression was screened during early embryonic development from fertilization (FE) to the 10-somite stage (10SS) by quantitative real-time PCR (qPCR). Class discovery analysis resulted in two main clusters containing 15 and 6 genes (Figure 2A). The relative expressions of the 15 genes, grouped together in the largest cluster, changed significantly during embryonic development (*p <*0.05). Inside this cluster, the four genes *askopos *(*kop*), *si:dkey-30j22.9*, *Tudor 5 protein *(*Tdrd5*) and HHC00130 (Stathmin family member) were sorted into a sub cluster showing very low to zero expression during the later stages of development. This was confirmed by the significant change in relative expression among the developmental stages (*kop*: *F*_6,28 _= 189.9, *p <*0.001; *si:dkey-30j22.9*: *F*_6,28 _= 28.9, *p <*0.001; *Tdrd5*: *F*_6,28 _= 74.5, *p <*0.001; HHC00130: *F*_6,28 _= 62.1, *p *< 0.001) as shown in Figure 2B; B, I, and 2B; L. For these four maternal genes their relative expression decreased significantly to a very low or zero level of expression between the blastula stage (BL) and the germ ring stage (GR). A similar significant drop between these two stages was found for six other genes in this cluster (HHC00068: *F*_6,28 _= 4.0, *p *< 0.001; HHC00309: *F*_6,28 _= 8.4, *p *< 0.001; HHC00334: *F*_6,28 _= 3.0, *p *< 0.02; HHC01010: *F*_6,28 _= 1.7, *p *< 0.001; HHC01032: *F*_6,28 _= 13.5, *p *< 0.001; HHC01310: *F*_6,28 _= 14.8, *p *< 0.001) (Figure 2B; K, O, P, Q, S, and 2B; U) though these did not decrease to similar low or zero relative expression during later stages. The remaining five genes of this larger cluster did not show any significant difference in gene expression between the blastula and germ ring stage.

The smaller cluster contained genes showing an opposite expression pattern (Figure 2A). In this group of six genes, lower relative expression during early developmental stages was observed in comparison to the later stages. The expression of the three maternal-zygotic genes *prohibitin 2 *(*phb2*), HHC00057 (orthologue of cullin) and HHC00255 (orthologue of phosphoinositide-dependent kinase 1) (Figure 2B; E, J and 2B; N) increased significantly from the FE stage to the 10SS stage (*phb2*: *F*_6,28 _= 9.0, *p *< 0.001; HHC00057: *F*_6,28 _= 7.8, *p *< 0.001; HHC00255: *F*_6,28 _= 4.0, *p *< 0.005). Two of the genes in this cluster did not show any significant differences in relative gene expression during early embryonic development (Figure 2B; *HR6A*, H and HHC00222, M). Gene expression between the five batches of Atlantic halibut eggs that were analyzed was found to be significantly different (*p *< 0.05) for all genes except from *HR6A *and HHC00222.

### Relative gene expression of maternal transcripts in relation to egg quality

**Figure 3:**
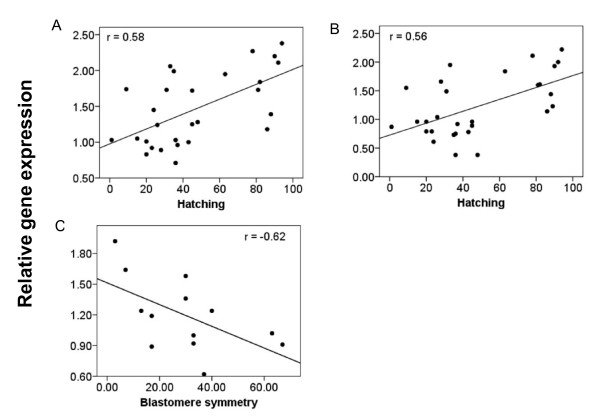
**Correlations between gene expression and egg quality (*r *> 0.5)**. Gene expression in relation to hatching (%): (A) HHC00353 (*n *= 29) and (B) HHC01517 (*n *= 29). Gene expression in relation to normal blastomer symmetry (%): (C) HHC00255 (*n *= 13). The correlation coefficient (*r*) is given for each regression line in each plot.

The relative gene expression of 18 genes was measured at the 8-cell stages in 29 different batches of Atlantic halibut eggs (Additional file 2). No significant correlations between gene expression and fertilization rates were found. The expression of seven genes (39% of total) correlated positively with hatching rates. The two genes HHC00353 and HHC01517 correlated stronger with hatching (*r *> 0.5, HHC00353: *r *= 0.58, *p *< 0.01 and HHC01517: *r *= 0.56, *p *< 0.01) (Figure 3A and 3B), compared to the other five genes (*r *≤ 0.5, *kop*: *r *= 0.38, *p *< 0.05; *si:dkey-30j22.9*: *r *= 0.50, *p *< 0.05; HHC0057: *r *= 0.41, *p *< 0.05; HHC00130: *r *= 0.43, *p *< 0.05; HHC00255: *r *= 0.41, *p *< 0.05) (Additional file 7) The expression of HHC00255 negatively correlated with the percentage of symmetric blastomeres (*r *= -0.62, *p *< 0.05) (Figure 3C).

## Discussion

### Characterization of the EST library

1419 maternal ESTs were previously reported in Atlantic halibut from sequencing an unbiased cDNA library obtained from 2-cell stage embryos [16]. In the present study we used suppressive subtractive hybridization (SSH) to subtract the transcripts expressed both before and after the switch from maternal to zygotic expression. The analysis resulted in a library containing 2341 EST sequences. Due to the relatively short size of the ESTs, the default cut-off <10-3, recommended by the software PartiGene, was chosen for the BLASTX analysis [20]. This decreased the stringency of the search, decreasing the possibility that significant matches would be overlooked. The low redundancy of the library of 2.7 suggests that the SSH worked efficiently since 77% of the putative genes were singletons containing only one EST, representing rare mRNAs. However, the largest gene clusters encoded for common genes such as cytochrome *b *and cytochrome oxidase subunits (Table 1). Annotated genes in the previous study contained almost twice as many genes classified as involved in metabolic processes compared to our library. In addition, genes involved in developmental processes were almost absent representing only 0.1% of the total genes compared to our library with 8% (Figure 1).

### Screening of relative gene expression during early embryonic development

The three genes: *kop*, *si:dkey-30j22.9 *and *Tdrd5 *showed an expression pattern typical for maternal genes (Figure 2B; A, B and 2B; I). By stage 25% epiboly (25EB), their expression was not detectable anymore, possibly due to the degradation of their transcripts. This could indicate that the MZT in Atlantic halibut takes place between the BL and GR stage. *kop *mRNA is continuously expressed in the zebrafish primordial germ cells (PGCs) during migration towards the putative gonads [27]. *Si:dkey-30j22.9 *and *Tdrd5 *encode proteins containing several Tudor domains. Tudor domains were identified as common protein motifs found in the *Drosophila *Tud protein which plays a dual role in abdomen development and germ cell formation [28,29]. *Si:dkey-30j22.9 *encodes an uncharacterized protein found in the zebrafish containing 6 tudor domains. *Tdrd5 *has been found to be expressed exclusively in mouse testis, implying that expression of this gene is restricted to the male germ line throughout development to adulthood [30]. It is unknown how *kop*, *si:dkey-30j22.9 *and *Tdrd5 *may influence embryonic development.

The three genes *phb2*, HHC00057 and HHC00225 were significantly up-regulated during the later embryonic stages, representing maternal-zygotic genes (Figure 2B; E, J and 2B; N). *phb2*, together with *prohibitin 1 *(*phb1*), codes for highly conserved proteins in eukaryotic cells that are present in multiple cellular compartments. In rainbow trout, *phb2 *mRNA abundance was found to correlate negatively with developmental success [31]. HHC00057 codes for a Cullin protein orthologue. They are RING H2 finger proteins that are part of a protein complex which forms the largest known class of ubiquitin ligases, the cullin-RING ubiquitin ligases (CRLs). In zebrafish, CUL2 has been found to be required for normal embryonic development and vasculogenesis [32]. HHC00255 codes for an orthologue of 3-phosphoinositide-dependent protein kinase-1 (PDPK1) which mediates the cellular effect of insulin and growth factors by activating a group of kinases [33,34]. It also plays a role in cell cycle resumption during oocyte maturation in starfish [35]. Lawlor et al. showed that PDPK1-deficient mice embryos displayed multiple abnormalities including lack of somites, forebrain and neural crest derived tissues and died after a few days. Mice embryos with reduced PDPK1 activity were 40-50% smaller than normal animals. The volume of a number of PDPK1-deficient cells got reduced by 35-60%, but not their cell number, nuclear size or proliferation [36]. How PDPK1 influences fish embryonic development has not been studied yet.

### Relative gene expression of maternal transcripts in relation to egg quality

In this study on Atlantic halibut we related maternal transcript levels with hatching success and normal blastomer symmetry. We used egg batches from different breeders, held under different feeding conditions and photoperiods, collected during three years. Because of the heterogeneous origin of the eggs, we were able to study gene expressions in relation to egg quality parameters in general. Correlations were significant but low, due to the heterogeneous origin of the eggs. The two genes HHC00353 and HHC01517 correlated stronger with hatching (*r *> 0.5,  *p *< 0.01) (Figure 3A and 3B), compared to five other genes, *kop*, *si:dkey-30j22.9*, HHC00057, HHC00130 and HHC00255) (Additional file 7) which showed statistically significant but very minor correlations. The expression of HHC00255 negatively correlated with the percentage of symmetric blastomeres (*r *> -0.5,  *p *< 0.05) (Figure 3C).

HHC00353 codes for an orthologue of an exportin 1-like protein. It is a member of the importin β superfamily of nuclear transport receptors. XPO1, also known as CRM1, is a major receptor for the export of proteins and RNAs out of the nucleus. XPO1 is also implicated in various steps during mitosis [37]. In the African clawed frog (*Xenopus laevis*) inhibitions of XPO1 activity was leading to a developmental arrest during neurulation [38]. The role of XPO1 in embryonic development in fish has not been studied. HHC01517 encodes a protein with Bric-a-brack, Tramtrack and Broad-complex (BTB) domains. In *Drosophila*, the maternally expressed gene *pipsueak *(*psq*) codes for a BTB domain protein (PsqA) which is required for correct abdominal segmentation in embryos but it is unknown if it plays a similar role during embryogenesis in fish [39]. The relation of *kop*, s*i:dkey-30j22.9 *and HHC00057 to embryonic development has been described above. HHC00130 encodes for an orthologue to the stathmin protein family. In zebrafish, the temporal and spatial expression of two orthologues of *STMN2 *has been described [40], although it is not known whether *STMN2 *has a regulatory role during embryonic development. Expression of HHC00255 was found to be positive correlated to hatching percentage and negative correlated with normal blastomer symmetry. This is in contrast to earlier findings during embryonic development in mice, where a reduced expression of PDPK1 was found to influence cell size [36]. However, abnormal blastomer symmetry is defined not only as cells of unequal size, but also by asymmetric cell positioning, incomplete inter-cell adhesion, poorly defined cell margins and vascuolar inclusions between cells [17]. HHC00255 high expression levels could be an indicator for suboptimal regulation of pathways involved in growth and/or cell division during early cell division. In several marine fish species, early cell symmetry has been found to correlate with high hatching and survival rates [41-43]. In contrast, others argued that high hatching and survival rates are maintained through cell symmetry corrections in consecutive developmental stages [44-46]. None of these studies have investigated the molecular mechanisms that regulate early cell divisions. In this study we have, for the first time, found a correlation between gene expression and blastomere symmetry. With 67% being the highest percentage of eggs with normal blastomere symmetry, symmetry was generally low in this study. Even though Shields et al established a score system for blastomere symmetry, the method suffers from its subjectivity [17]. It is unclear if the estimated low blastomere symmetry in this study were true or due to a too strict estimation of symmetry. *phb2 *has earlier been shown to be differentially expressed in eggs with low and high developmental potential in rainbow trout [31]. In the present study, expression levels of *phb2 *did differ significantly in eggs with low and high hatching rates and did not correlate with hatching success.

In this study, poor hatching success was not correlated with a general decrease in transcript abundance but with low transcript levels for specific genes. Similar gene specific relations were found when Aegerter et al [7] studied the relation of maternal genes and eyed-stage survival in rainbow trout eggs. Out of the seven studied genes they found three genes to be down-regulated and four to be up-regulated in eggs with low survival compared to eggs with high survival. In the same study, similar variations in gene expression were found in post-ovulatory eggs. Thus, the low expression levels in Atlantic halibut low quality eggs cannot be entirely explained by the post-ovulatory aging of eggs. A possible explanation could be a reduced incorporation of specific maternal mRNAs into the eggs during oogenesis. Furthermore, maternal mRNAs in the oocytes are usually activated and stabilized by polyadenylation before translation. Failed polyadenylation can trigger degradation and translation repression, resulting in low expression levels and poor embryonic development [47]. In a previous single pair's cross study in Atlantic halibut, a significant but weak paternal effect on fertilization and hatching was found compared to the maternal effect [48]. In human, sperm cells do not only deliver the haploid genome but also mRNA and small sperm RNA molecules that might interfere in gene expression (iRNA) [49]. By using two instead of one male we tried to reduce the paternal effect but a certain influence cannot be excluded.

## Conclusions

During the last decade there has been a notable increase in genomic resources available for species of commercial interest for aquaculture. Nevertheless, the application of this information to aquaculture is still poor. In this study, we have for related levels of gene expression to hatching success in a commercial species, Atlantic halibut. First, we increased the available genomic information of maternal Atlantic halibut genes by constructing an EST library. By screening a selection of genes during early development we characterized the expression of maternal and maternal-zygotic genes. Finally we related gene expression of maternal transcripts to Atlantic halibut egg quality. Poor hatching success was not correlated with a general decrease in transcript abundance but with low transcript levels of specific genes. Thus, low Atlantic halibut egg quality cannot be entirely explained by post-ovulatory aging.

Future functional studies on these genes will be useful to identify the molecular mechanism related to egg quality and developmental success in Atlantic halibut.

## Competing interests

The authors declare that they have no competing interests.

## Authors' contributions

MM and SLB performed the sampling and estimation of egg batch parameters. MM constructed the cDNA library, carried out bioinformatics analysis with JMOF, and performed the qRT-PCR together with TTB. MM wrote the manuscript. JMOF, IB and IAJ planned the research and edited the manuscript. All authors read and approved the manuscript.

## Supplementary Material

Additional file 1 **List of genes used for quantification of gene expression of Atlantic halibut maternal library**. Genes with BLASTX hits are given first, followed by genes without BLAST hits. For each gene, BLASTX hit result or conserved domain search results are given together with species name, accession number, E-value, and gene function if available. References are given for genes with documented roles in early development. Indications are given in which qPCR experiments genes were used. Experiment 1: Expression from fertilization to the 10-somite stage, Experiment 2: Expression at the 8-cell stage in 29 different egg batches. Click here for file

Additional file 2 **Sample overview of Atlantic halibut egg batches**. Samples were collected from fifteen female Atlantic halibut at two locations, Bodø University College (1) and Risørfisk AS (2). For each female, weight, photoperiod: Natural photoperiod (N) or advanced photoperiod (A) are given. For each batch, sample year, batch number, incubation method: Small-scale in Petri-dishes (S) or large-scale in 280 l incubators (L) are given. For each batch incubated in small-scale, fertilization rate (% ± SD, *n *= 3), hatching rate (% ± SD, *n *= 3) and rate of symmetric blastomeres (% ± SD, *n *= 30) are given. n.a stands for blastomere symmetry not evaluated. Click here for file

Additional file 3 **Primer information of selected genes**. For each reference gene, primer sequences, amplicon sizes, reaction efficiencies (E) and Pearson's coefficients of determination (R^2^) are shown. Click here for file

Additional file 4 **Genes used for reference genes determination and their primer information**. The gene name, gene symbol, accession number and function are shown. For each primer pair, sequences, amplicon sizes, reaction efficiencies (E) and Pearson's coefficients of determination (R^2^) are shown. Click here for file

Additional file 5 **Reference gene stability values**. Ranking of reference genes according to their expression stability throughout early embryonic development (A) and in twenty-nine batches of egg at the 8-cell stage (B). Average expression stability values were calculated by *geNorm*. Expression stability of the reference genes is inversely correlated to their stability index. Click here for file

Additional file 6 **Ct values for embryonic development reference genes**. The raw cycle thresholds (Ct ± SE) data for the reference genes used to normalize relative expression during embryonic development (*n *= 5). A: HHC00353 and B: HHC01517. Click here for file

Additional file 7 **Correlations between gene expression and egg quality (*r *≤ 0.5)**. Gene expression in relation to hatching (%): (A) *kop*, (B) *si:dkey-30j22.9*, (C) HHC00057, (D) HHC00130 and (E) HHC00255) (*n *= 29). The correlation coefficient (*r*) is given for each regression line in each plot. Click here for file
