# Supplementary material for: Maternal gene expression in Atlantic halibut (Hippoglossus hippoglossus L.) and its relation to egg quality
Source: BMC Res Notes. 2010 May 24;3:138. doi: 10.1186/1756-0500-3-138 (PMC2897799; doi:10.1186/1756-0500-3-138)
Supplement: Additional file 1 — List of genes used for quantification of gene expression of Atlantic halibut maternal library. Genes with BLASTX hits are given first, followed by genes without BLAST hits. For each gene, BLASTX hit result or conserved domain search results are given together with species name, accession number, E-value, and gene function if available. References are given for genes with documented roles in early development. Indications are given in which qPCR experiments genes were used. Experiment 1: Expression from fertilization to the 10-somite stage, Experiment 2: Expression at the 8-cell stage in 29 different egg batches. [file 1756-0500-3-138-S1.PDF]

Additional file 1

Selected genes					
**Gene name (Gene symbol)**	**BLASTX results (Species)**	**Accession number**	**E value**	**Description [Reference]**	**qPCR experiment**
Askopos (*kop*)	Askopos (*Danio rerio*)	Q5YCX2	6e-14	Expressed in germ plasma and primordial germ cells	1,2
Si:dkey-30j22.9	Si:dkey-30j22.9 (*Danio rerio*)	XM_688932.3	6e-37	Uncharacterized protein [19,20]	1,2
Betaine aldehyde dehydrogenase (*BAH*)	Betaine aldehyde dehydrogenase (*Oryzias latipes*)	NM_001104848.1	2e-32	Catalyzes Betaine aldehyde + NAD(+) + H(2)O <=> Betaine + NADH	1
Checkpoint 1	CHK1 checkpoint homolog (*Xenopus tropicalis*)	CR848200.2	3e-17	Regulates mitosis	1
Prohibitin 2 (*phb2*)	Prohibitin-2 (*Salmo salar*)	NM_001141404.1	5e-30	Chaperone-like regulator of AAA protease [22]	1,2
Synthaxin 4	Syntaxin 4 (*Lateolabrax japonicus*)	EF513752.1	1e-38	t-SNARE protein receptor	1
18K hypothetical goldfish protein (*18k Gold*)	Hypothetical 18K protein goldfish mitochondrion (*Orconectes australis*)	JC1348	2e-17	Uncharacterized protein	1

Table continues on next page

HR6A	Predicted similar to ubiquitin-conjugating enzyme HR6A (*Ornithorhynchus anatinus)*	XM_001511341.1	1e-31	DNA damage resistance	1
Tudor 5 protein (*Tdrd5*)	Tdrd5 protein (*Danio rerio*)	BC134985.1	1e-12	Formation of primordial germ cells and normal abdominal segmentation [19,20]	1,2
HHC00005	Profilin 2 like protein (*Danio rerio*)	CAN88191	3e-06	Profilin binds actin monomers, membrane polyphosphoinositides and poly-L-proline.	2
HHC00036	Unnamed protein product	CAF92632	7.2	Uncharacterized protein	2
HHC00106	Creatine kinase 1	ABU42561	2e-102	Catalysis conversion from creatin to phosphocreatin	2
HHC00223	RNA binding protein with multiple splicing 2 (*Danio rerio*)	NP_956553	1e-59	RNA binding protein	2
HHC00189	Similar to Lamina-associated polypeptide 2 isoform alpha (*Danio rerio*)	XP_001921942	3e-16	Cell cycle progression	2
HHC00353	Exportin-1	XP_870795	6e-42	Nuclear transport receptor [31]	2
HHC01032	Unnamed protein product	CAG12058	5e-53	Uncharacterized protein	2
HHC01306	SH3-domain GRB2-like endophilin B2 (*Danio rerio*)	CAM15470	1e-73	Protein binding	2

Table continues on next page

HHC01385	Caprin family member 2 (*Danio rerio*)	NP_001013291	4e-20	Regulates transport and translation of mRNAs of proteins involved in synaptic plasticity in neurons and cell proliferation and migration in multiple cell types	2
HHC01481	Hypothetical protein LOC561007 (*Danio rerio*)	NP_001122176	8e-70	Uncharacterized protein	2

**Gene name**	**Conserved domain search results**	**E value**	**Description**	
HHC00057	Cullin	7e-04	A subunit of E3 ubiquitin [23]	1
HHC00068	Elongin subunit A	2e-10	RNA polymerase II transcription factor SIII	1
HHC00130	Stathmin family	9e-16	Regulation of the microtubule cytoskeleton [32]	1,2
HHC00222	SET domain	1e-12	Protein lysine methyltransferase enzymes	1
HHC00255	Phosphoinositide-dependent kinase 1	5e-50	Regulates cell volume [26,27]	1,2
HHC00309	JmjC domain	2e-28	Histone modification	1
HHC00334	CDC45-like protein	4e-60	DNA replication	1
HHC01010	Dynamin family	1e-05	GTPases	1

Table continues on next page

HHC01015	BTG family	8e-08	Antiproliferative factors. regulation of cell growth and cell differentiation	1
HHC01032	SET domain.	2e-21	Protein lysine methyltransferase enzymes	1
HHC01194	Geminin	2e-10	Inhibits DNA replication	1,2
HHC01310	Tetratricopeptide repeat domain	2e-4	Variety of functions including protein-protein interactions	1
HHC01517	BTB domain	2e-35	Protein-protein interaction	2
