# Supplementary material for: Maternal gene expression in Atlantic halibut (Hippoglossus hippoglossus L.) and its relation to egg quality
Source: BMC Res Notes. 2010 May 24;3:138. doi: 10.1186/1756-0500-3-138 (PMC2897799; doi:10.1186/1756-0500-3-138)
Supplement: Additional file 2 — Sample overview of Atlantic halibut egg batches. Samples were collected from fifteen female Atlantic halibut at two locations, Bodø University College (1) and Risørfisk AS (2). For each female, weight, photoperiod: Natural photoperiod (N) or advanced photoperiod (A) are given. For each batch, sample year, batch number, incubation method: Small-scale in Petri-dishes (S) or large-scale in 280 l incubators (L) are given. For each batch incubated in small-scale, fertilization rate (% ± SD, n = 3), hatching rate (% ± SD, n = 3) and rate of symmetric blastomeres (% ± SD, n = 30) are given. n.a stands for blastomere symmetry not evaluated. [file 1756-0500-3-138-S2.PDF]

Additional file 2

Female	Location	Weight	Photo-period	Sample year	Batch nr.	Incubation	Fertilization (%)±SD *n* = 3	Hatching (%)±SD *n* = 3	Symmetry (%)±SD *n* = 30
WF1	1	37	N	2008	1	S	91 ± 1	88 ± 1	n.a
					2		93 ± 1	86 ± 3	n.a
					3		92 ± 1	37 ± 9	n.a
					4		38 ± 3	26 ± 3	n.a
G39R	1	39	A	2008	1	S	49 ± 1	20 ± 2	n.a
					2		60 ± 8	36 ± 6	n.a
					3		35 ± 2	23 ± 9	n.a
					4		26 ± 5	15 ± 4	n.a
				2006	1	L	49 ± 1	33	33 ± 0
G39S	1	80	N	2009	1	S	68 ± 1	24 ± 2	37 ± 0
				2008	1		89 ± 3	43 ± 7	n.a
					2		13 ± 1	1 ± 1	n.a
G32	1	45	A	2008	1	S	25 ± 2	20 ± 1	n.a
G8	1	83	N	2009	1	S	69 ± 2	45 ± 1	30 ± 0
				2008	1		84 ± 3	28 ± 4	n.a
Y46	1	67	N	2009	1	S	80 ± 1	45 ± 1	63 ± 0
				2006	1	L	90 ± 3	81	57 ± 1
					2		90 ± 3	82	33 ± 0
Y32	1	36	A	2006	1	L	20 ± 2	9	17 ± 0
Y7	1	32	A	2006	1	L	43 ± 1	31	7 ± 0
O17	1	96	N	2006	1	L	74 ± 2	63	7 ± 0
					2		96 ± 5	89	37 ± 0
Y30	1	95	N	2009	1	S	86 ± 1	48 ± 2	13 ± 0
				2006	1	L	43 ± 4	35	3 ± 0
Y4	1	36	A	2009	1	S	84 ± 1	36 ± 1	40 ± 0
R1	2	30-40	N	2007	1	L	92 ± 2	92	n.a
R2	2	30-40	N	2007	1	L	93 ± 1	90	n.a
R3	2	30-40	N	2007	1	L	68 ± 2	78	n.a
R4	2	30-40	N	2007	1	L	90 ± 3	94	n.a
