# Supplementary material for: Maternal gene expression in Atlantic halibut (Hippoglossus hippoglossus L.) and its relation to egg quality
Source: BMC Res Notes. 2010 May 24;3:138. doi: 10.1186/1756-0500-3-138 (PMC2897799; doi:10.1186/1756-0500-3-138)
Supplement: Additional file 3 — Primer information of selected genes. For each reference gene, primer sequences, amplicon sizes, reaction efficiencies (E) and Pearson's coefficients of determination (R2) are shown. [file 1756-0500-3-138-S3.PDF]

Additional file 3

Name	Forward	Reverse	Size (bp)	E (%)	R^2
Askopos	TCTGGTAGTTCCTGCGTGTGAG	GCTCTTCAACCTCATCACCCA	55	104	0.998
Sidkey-30j22.9	GCAAGGTGTCACTCAAGGCAC	GTACTTCAGACCTGTGGAGGGTT	95	102	0.999
Betaine aldehyde dehydrogenase	GTATCCACCAAACGGCACTTC	GCAGGTACTCAGGCGAGCC	50	104	0.999
Checkpoint 1	GGCAGGTACTCATTCCAATTACAG	GAAACGGCTACCACATCCAAG	83	100	0.998
Prohibitin 2	GGAAGGACTACGACGAGCGAG	GGGACACCTGTGCTCTCTGTG	69	99	0.999
Synthaxin 4	GATGATGAAAATGAGGACAAAGC	CCCATCCTCCTCTGACTTCTTG	252	100	0.998
18K hypothetical goldfish protein	AGTTACTTCTTCTCCCGCAAGC	GATCCAACATCGAGGTCGTAAAC	122	93	0.999
HR6A	TATGTTTGGACATCCTACAGAATCG	CGGACTGTTGGGATTTGGTTC	58	98	0.999
Tudor 5 protein	CTGTCACTCTGAGGGCTTTATCC	TCTGCTGGATGTGGCTCCTC	88	100	0.997
HHC00057	CAGGTCGTCTGTTTTGCCATTC	CATAAAGAAGGTGGAAGCCAGG	146	92	0.999
HHC00068	ACATCTCCTCCCACGATTCA	TTGAGGAGTGCAACCCAATC	114	98	0.998
HHC00130	GGAGGGATCTTTGGTTTCTTTG	CAACAAGGAGAACCGCACAG	67	99	0.998
HHC00222	TACTGTGTAGATGCCACGAAAGAG	CCGTTGATGTCGTGGAGTTTG	96	97	0.999
HHC00255	ATAATACATCCCAAAGCCCAGAG	CACAATAAGGGGATAATACACAGAGA	198	104	0.999
HHC00309	GGACGGGGAGATTAGAGTCATC	GAGCCCAAGTCCTGGTATGCC	93	105	0.998
HHC00334	CCATGAGGTAGCAGTAGAGGAAGG	GCTGGTATTGTCCTGGCGAAG	115	95	0.997
HHC01010	GAAGAGAGGAAGAACATAAAGACGG	CATCCCTGAGTAGAGCACACTTG	149	93	0.999
HHC01015	CCACTAGAAGTGTGTGCAAGATC	CGTTTCCAGGTTTTTTGAATCC	85	97	0.998
HHC01032	CCGCATTGATGACTTTGATGTG	CTGGACTCATAGTGGCTAATTCACC	143	99	0.996

HHC01194	ACTAAGACCCAGCCAGCAGAAG	GGTGGAGGGAGGAGTTTCTTTG	179	99	0.999
HHC01310	TATGAGGAAGCGGTGGTTTG	GAGCCTGCCCAACCTTATCAT	75	98	0.998
HHC00005	CCGAGCGGAGGGATTTAGC	CAGCCATCAGGTTGTCCACG	229	99	0.998
HHC00036	TAAACGGCTCTGTTGTCCCAT	GTACACGCTGTCGCTTCCAG	284	92	0.999
HHC00106	AGGCATCTGGCACAATGAGAAC	CTCTCTACTTCTGGGCAGGGAT	462	89	0.999
HHC00189	GGGTGGCTTCATCTCACTTC	GTTTGAACCCGTGTCGGAG	313	95	0.998
HHC00233	AACAGCCTGTCGGGTTTGTAAC	GATGCTGGGATCAGTGCTGC	211	92	0.997
HHC01306	TCGGCTTTTTCCACTTCCTC	GGTGGGGGAATGTGAGAGAAG	262	98	0.999
HHC01032	CTATGATGGCAAGGGTATCGG	TTAGGGCTTACTAGCGAACGGG	297	99	0.998
HHC01385	GAACTACCAGAGGAGAGGGCAATC	CTTACCCAAAGACCCCATCCTG	127	98	0.997
HHC01481	GGGACTCATCTCGTGGGTCT	AGCAGTATTACTTGACCTCGCC	125	98	0.999
