# Supplementary material for: Maternal gene expression in Atlantic halibut (Hippoglossus hippoglossus L.) and its relation to egg quality
Source: BMC Res Notes. 2010 May 24;3:138. doi: 10.1186/1756-0500-3-138 (PMC2897799; doi:10.1186/1756-0500-3-138)
Supplement: Additional file 4 — Genes used for reference genes determination and their primer information. The gene name, gene symbol, accession number and function are shown. For each primer pair, sequences, amplicon sizes, reaction efficiencies (E) and Pearson's coefficients of determination (R2) are shown. [file 1756-0500-3-138-S4.PDF]

Additional file 4

Name (Abbrevation)	Accession number	Forward	Reverse	Size (bp)	E (%)	R^2
HHC01138	NP_989715	CAGTCCTGGCGACCGATGT	CAAGATGGAGATTCGCAACTGT	76	99	0.998
HHC00353	CAAE00000000.1	CGAGGTACTCTCCACTCTCATTCTC	AACCTCAGTTTTTATCCAGGTTCAC	81	98	0.999
HHC01517	NM_001099229	AGCAGGTTCTCCATGTTGAGTG	CTATTTCAAAGCCATGTTCACAGG	143	95	0.998
β2-Tubulin 2 (*Tubb2*)	DT805564	CTACAATGAGGCTTCAGGTGG	TCCCTCTGTGTAGTGACCCTTG	134	96	0.998
β-Actin (*Actb*)	EB103323	GAGAAGATGACTCAGATCATGTTCG	CCAGCCAGGTCCAGACGG	154	91	0.999
Elongation factor 2 (Eef2)	EB173938	ATGGAGTCATTTGGTTTCACAGC	GAGACCCTTGCGTTTGCG	121	94	0.999
