# Supplementary figures and images for: Maternal gene expression in Atlantic halibut (Hippoglossus hippoglossus L.) and its relation to egg quality
Source: BMC Res Notes. 2010 May 24;3:138. doi: 10.1186/1756-0500-3-138 (PMC2897799; doi:10.1186/1756-0500-3-138)

**A**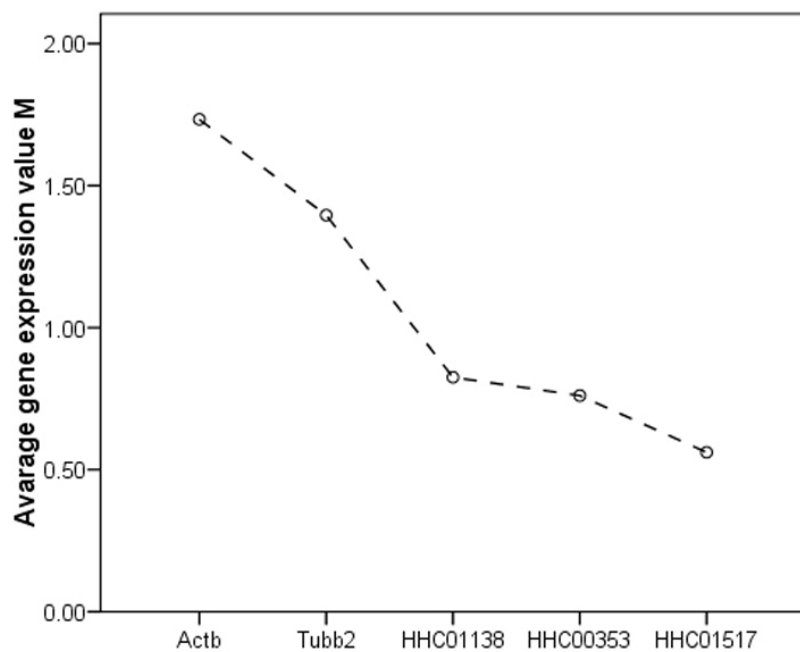**B**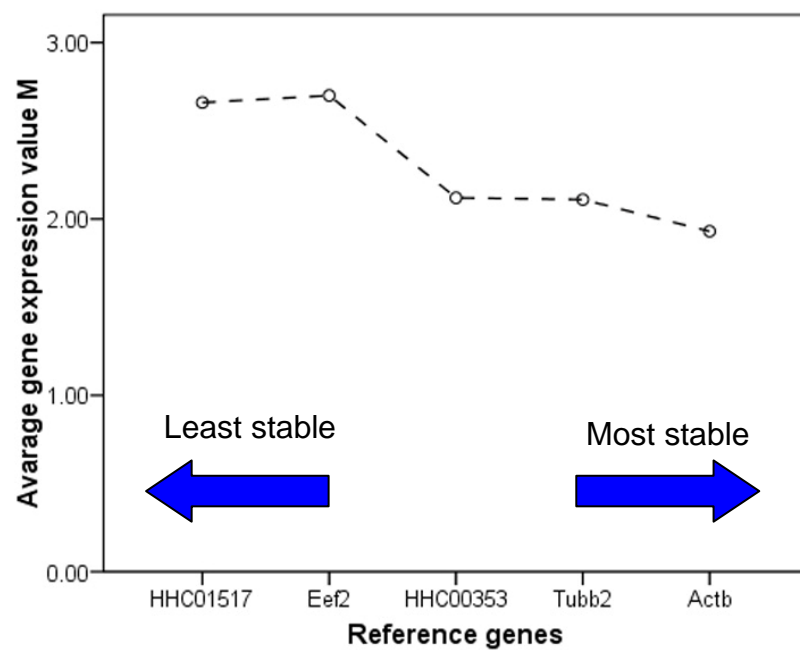

Supplement: Additional file 5 — Reference gene stability values. Ranking of reference genes according to their expression stability throughout early embryonic development (A) and in twenty-nine batches of egg at the 8-cell stage (B). Average expression stability values were calculated by geNorm. Expression stability of the reference genes is inversely correlated to their stability index. [file 1756-0500-3-138-S5.PDF]

A

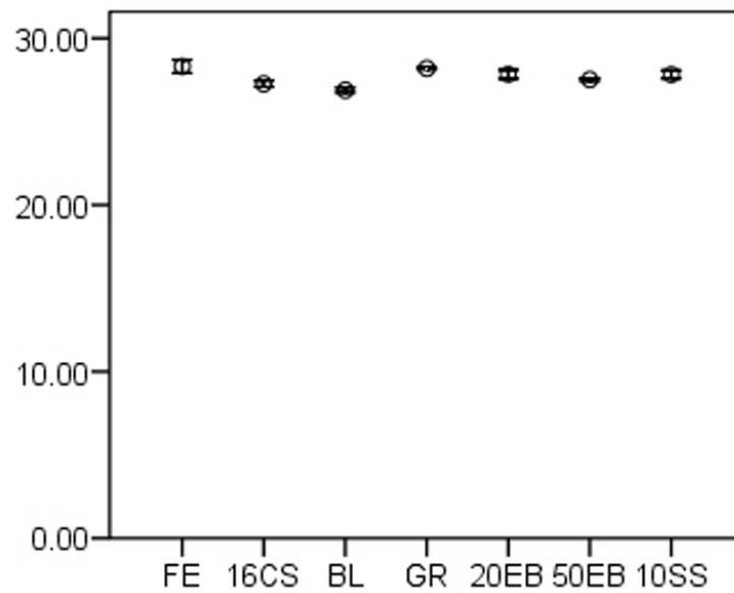

B

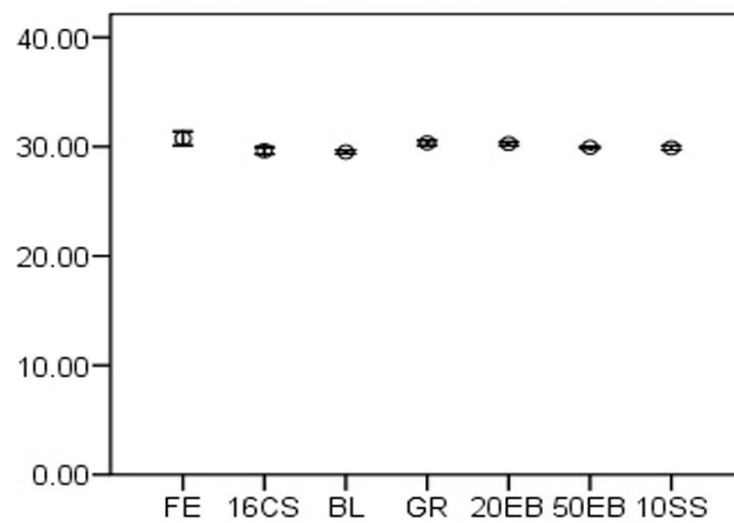

Developmental stages

Supplement: Additional file 6 — Ct values for embryonic development reference genes. The raw cycle thresholds (Ct ± SE) data for the reference genes used to normalize relative expression during embryonic development (n = 5). A: HHC00353 and B: HHC01517. [file 1756-0500-3-138-S6.PDF]

Relative gene expression

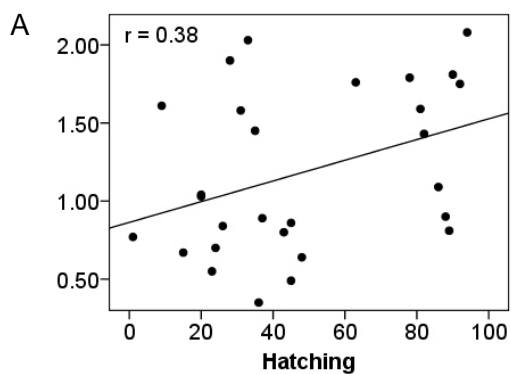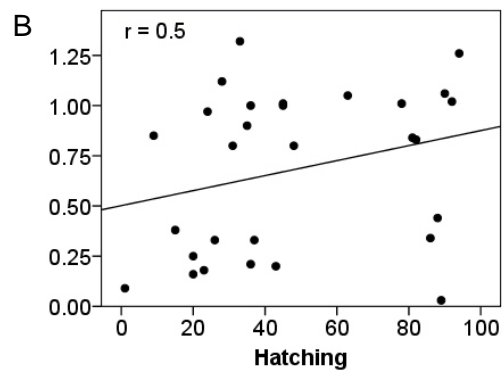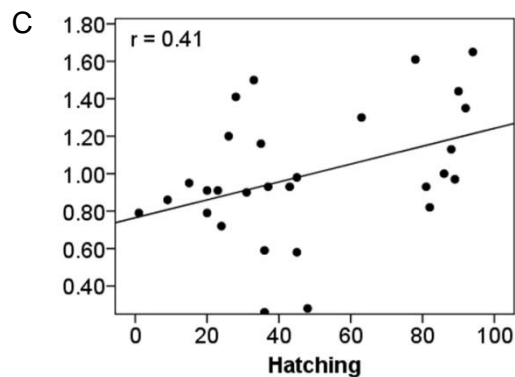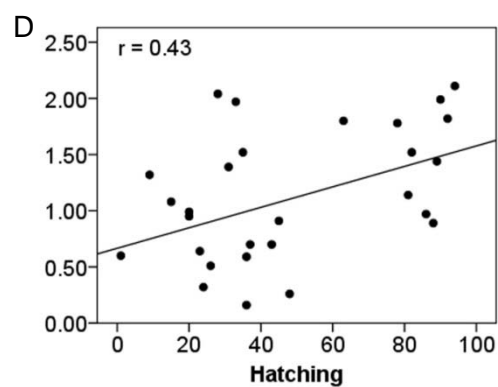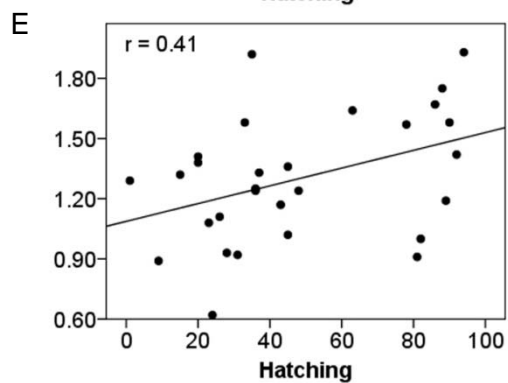

Supplement: Additional file 7 — Correlations between gene expression and egg quality (r ≤ 0.5). Gene expression in relation to hatching (%): (A) kop, (B) si:dkey-30j22.9, (C) HHC00057, (D) HHC00130 and (E) HHC00255) (n = 29). The correlation coefficient (r) is given for each regression line in each plot. [file 1756-0500-3-138-S7.PDF]
